# Increased Stability and Breakdown of Brain Effective Connectivity During Slow-Wave Sleep: Mechanistic Insights from Whole-Brain Computational Modelling

**DOI:** 10.1038/s41598-017-04522-x

**Published:** 2017-07-05

**Authors:** Beatrice M. Jobst, Rikkert Hindriks, Helmut Laufs, Enzo Tagliazucchi, Gerald Hahn, Adrián Ponce-Alvarez, Angus B. A. Stevner, Morten L. Kringelbach, Gustavo Deco

**Affiliations:** 1 Center for Brain and Cognition, Computational Neuroscience Group, Universitat Pompeu Fabra, Calle Ramón Trias Fargas 25-27, 08005 Barcelona, Spain; 2 Department of Neurology, Christian Albrechts University Kiel, 24104 Kiel, Germany; 3 Department of Neurology, UKSH, Arnold-Heller-Straße 3, 24105 Kiel, Germany; 4 Netherlands Institute for Neuroscience, Amsterdam-Zuidoost, Netherlands; 5 Department of Psychiatry, University of Oxford, Oxford, UK; 6 Center of Music in the Brain (MIB), Clinical Medicine, Aarhus University, Aarhus, Denmark; 7 Institució Catalana de la Recerca i Estudis Avançats (ICREA), Passeig LLuís Companys 23, Barcelona, 08010 Spain; 8 Department of Neuropsychology, Max Planck Institute for Human Cognitive and Brain Sciences, 04103 Leipzig, Germany; 9 School of Psychological Sciences, Monash University, Melbourne, Clayton VIC 3800 Australia

## Abstract

Recent research has found that the human sleep cycle is characterised by changes in spatiotemporal patterns of brain activity. Yet, we are still missing a mechanistic explanation of the local neuronal dynamics underlying these changes. We used whole-brain computational modelling to study the differences in global brain functional connectivity and synchrony of fMRI activity in healthy humans during wakefulness and slow-wave sleep. We applied a whole-brain model based on the normal form of a supercritical Hopf bifurcation and studied the dynamical changes when adapting the bifurcation parameter for all brain nodes to best match wakefulness and slow-wave sleep. Furthermore, we analysed differences in effective connectivity between the two states. In addition to significant changes in functional connectivity, synchrony and metastability, this analysis revealed a significant shift of the global dynamic working point of brain dynamics, from the edge of the transition between damped to sustained oscillations during wakefulness, to a stable focus during slow-wave sleep. Moreover, we identified a significant global decrease in effective interactions during slow-wave sleep. These results suggest a mechanism for the empirical functional changes observed during slow-wave sleep, namely a global shift of the brain’s dynamic working point leading to increased stability and decreased effective connectivity.

## Introduction

One of the great challenges in neuroscience is to understand the underlying mechanisms taking place in different conscious brain states. Sleep is a reversible state characterised by unresponsiveness and altered consciousness, distinguished from wakefulness by a decrease in the ability to interact with the external world^1^. A long line of sleep research, using primarily EEG, underlies our current classification of sleep into rapid eye movement (REM) and non-REM sleep. Current consensus further sub-divides non-REM sleep in three stages: N1, N2, and N3, where N3 is often referred to as slow-wave sleep^2^.

From a behavioural point of view the contrast between sleep and wakefulness is clear. However, while the EEG shows clear changes between the two, it is less clear how the brain’s spatiotemporal dynamics supports these different behavioural states. From this perspective recent decades’ research has improved our understanding of wakefulness in particular, characterising the organisation of the brain’s spontaneous activity in terms of correlated activity patterns across different brain regions (as measured with functional resonance imaging [fMRI]), commonly known as ‘resting-state networks’^3–8^. Using ICA and seed-based methods has found that resting-state networks appear to be preserved during sleep, even during slow-wave sleep^9–13^. Specifically, the default mode network (DMN) has been shown to be retained during slow-wave sleep^11^ albeit with altered connectivity strength and relations to other networks^12, 14^. More generally, slow-wave sleep is associated with a general decrease of cortico-cortical functional connectivity^12, 14–17^, and a diminished level of information integration^10, 11, 17, 18^. The repertoire of functional brain connectivity is constrained by the underlying anatomical connectivity^19^, and functional connectivity is more correlated to the anatomical backbone during states of deep sleep and anaesthesia compared to wakefulness^20, 21^.

Furthermore, combined transcranial magnetic stimulation and electroencephalography (TMS-EEG) studies have shown decreased cortical effective connectivity during deep sleep, with effective connectivity understood here as the capacity for a causal interaction in response to an external perturbation^22–25^. This result suggests a lowering of the capability of the brain to integrate information across different cortical areas and a diminished capacity to amplify local perturbations.

These major alterations during deep sleep suggest a change in collective brain dynamics compared to wakefulness, raising the challenge of providing a mechanistic understanding of the empirical observations based on qualitative changes in the local underlying dynamics of the brain.

To address this problem, we first study differences between wakefulness and deep slow-wave sleep by analysing functional connectivity and phase synchrony in an fMRI data set consisting of 18 participants falling asleep in the scanner. We apply a whole-brain computational model based on the normal form of a supercritical Hopf bifurcation incorporating underlying brain dynamics and unfolding over realistic brain anatomical connectivity^26^. This novel model is able to describe the transition from a stable focus presenting noisy oscillations to a stable limit cycle defined by fully sustained oscillations, and can characterise global brain dynamics in terms of their stability and global coupling. We investigate how these parameters change between wakefulness and slow-wave sleep by estimating them from the empirical data and identifying the optimal dynamic working point of each brain state. Finally, to identify the actual level of interaction between different brain nodes, we investigate the differences between the two brain states in terms of their effective connectivity based on the previously mentioned whole-brain model. This approach allows us to find a possible underlying mechanism explaining the empirically observed phenomena.

## Results

We investigated the differences between two different vigilance states: an awake resting-state condition and a slow-wave sleep condition in a group of 18 healthy human participants. We applied a data analysis approach and, to gain further insight, a whole-brain modelling approach in order to examine the differences between the two states in functional connectivity, synchrony, metastability and dynamic working region. Furthermore we analysed the effective connectivity in both conditions using whole-brain modelling.

### Lowering of functional connectivity, phase synchrony and metastability in deep sleep

**Figure 1:**
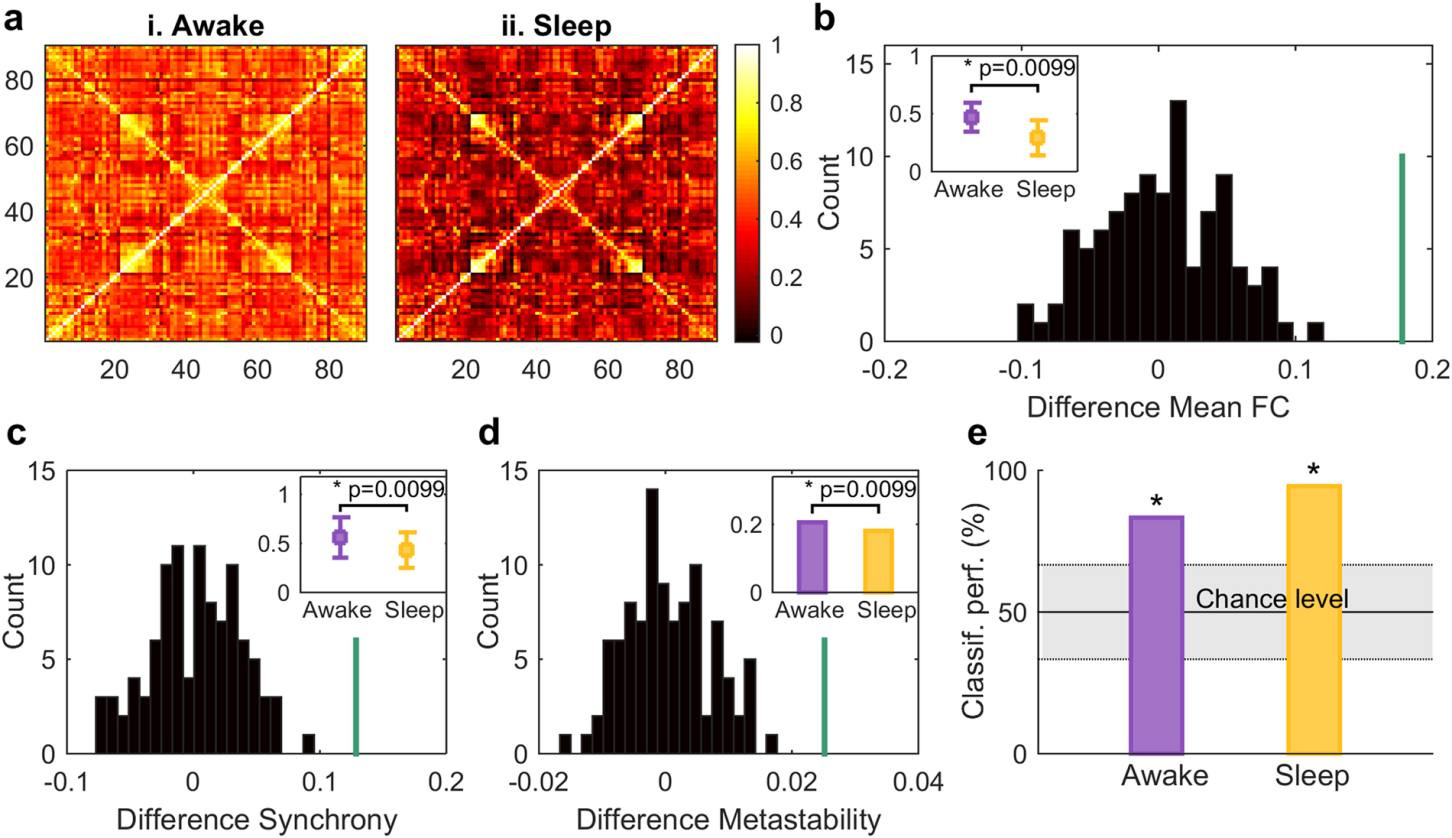
Data Analysis. In (**a**) the functional connectivity matrices averaged over participants are shown for the two different vigilance states. In (**b**) the difference of the mean of the upper triangle FC matrices (group average) between the awake and the sleep state are shown. The histogram (black) represents the distribution of the test-statistic under the null-hypothesis of no difference between vigilance states, whereas the green line shows the difference of the means of the empirical FC matrices. In the upper left corner the mean FCs and their standard deviations are shown. They are significantly different with a p-value of 0.0099. (**c**) demonstrates the difference between the mean synchrony in the awake state and the sleep state. The synchrony is calculated as the mean Kuramoto order parameter (see *Methods*). Again, the histogram represents the distribution of the test-statistic and the green line the difference between the synchrony in awake and sleep obtained from the empirical data. In the upper right corner the means and the standard deviations are shown, the mean synchrony is significantly higher in awake than in sleep with a p-value of 0.0099. In (**d**) the difference between the metastability in awake and in sleep is shown. The metastability is calculated as the standard deviation of the Kuramoto order parameter (see *Methods*). The histogram and the green line are to be read as in (**b**) and (**c**). In the upper right corner the metastability in awake and in sleep is represented, also in this modality a significant difference (p-value 0.0099) can be observed. In (**e**) the classification performance using a Gaussian classifier is shown for awake (violet) and sleep (yellow) test sets, respectively (see *Methods*). The vigilance state is predicted with high accuracy (83.33% and 94.44% for awake and sleep test sets, respectively) exceeding the 95th percentile of chance level.

We analysed the differences between the awake and the sleep state in functional connectivity, phase synchrony and metastability. We calculated the functional connectivity matrices for the two vigilance states by averaging the matrices of the Pearson correlations between the BOLD signals of all pairs of regions of interest (ROIs) over subjects within one vigilance group. This resulted in two FC matrices, one for each condition. Then, for comparing the two vigilance states, we computed the mean and standard deviation of both of the FC matrices and finally subtracted the mean FC value of the sleep state from the awake state. We found that the difference between the mean FC values in the two conditions was significantly higher than the differences found in the surrogate data created by randomly shuffling vigilance state assignments (awake: 0.471 ± 0.125, sleep: 0.293 ± 0.152; p-value: 0.0099) (Fig. 1b). The surrogate data we applied was constructed under the null-hypothesis of no difference between conditions, hence appropriate for comparing the two states amongst each other. Additionally, in order to verify that the group FC matrices are indeed representative of the average subject, we calculated the node strength for each individual FC matrix. We found that 83% of all the subjects exhibit higher FC node strength during wakefulness than during sleep (see Supplementary Fig. S1a), indicating that the group FC matrices indeed represent the average participant with high accuracy.

Next, in order to quantify the global level of synchronisation between the signals of each of the nodes, we computed the Kuramoto order parameter, a temporal measure taking values from 0 to 1, where 0 represents complete phase asynchrony and 1 complete synchronisation. Consequently we calculated the temporal mean and standard deviation of the Kuramoto order parameter and averaged over all subjects within one vigilance state group. We termed these two measures synchrony and metastability, respectively (see *Methods*). We found significantly higher synchrony in awake than in sleep (awake: 0.560, sleep: 0.431; p-value: 0.0099) (Fig. 1c) and significantly higher metastability in awake than in sleep (awake: 0.206, sleep: 0.181; p-value: 0.0099) (Fig. 1d).

### Classification of consciousness state with Gaussian classifier

We evaluated how specific the functional connectivity is to the vigilance state. We used a jackknife cross-validation procedure consisting of: first, calculating the covariances on a subset of the data from *N* − 1 participants, separately for each vigilance state, and then using these covariances to classify the data of the remaining subject (see *Methods*). We found that the Gaussian classifier significantly predicted the vigilance state with high accuracy (83.33% and 94.44% for awake and sleep test sets, respectively) (Fig. 1e). Thus, the whole-brain covariance of single participants reliably relates to the vigilance state, justifying the use of group measures.

Furthermore, we tested how similar the time-series of a single subject were to a random sample from the grouped time-series of the other *n*-1 subjects by using a similar procedure (see *Methods*). We expected the log-likelihood ratio to be approximately 1 if the time-series of the i-th subject were indistinguishable from a random sample taken from the time-series of the remaining subjects. We found that this was the case for most of the subjects: the log-likelihood ratio ranged between 0.67–1.52 and it was equal to 1.07 ± 0.13 and 1.00 ± 0.06 averaged over subjects, for awake data and sleep data, respectively (see Supplementary Fig. S1b). This result confirms that the fMRI time-series of single subjects strongly resemble the ones of the group and can be viewed as a random sample taken from it.

### Shift of the dynamic working point during sleep

**Figure 2:**
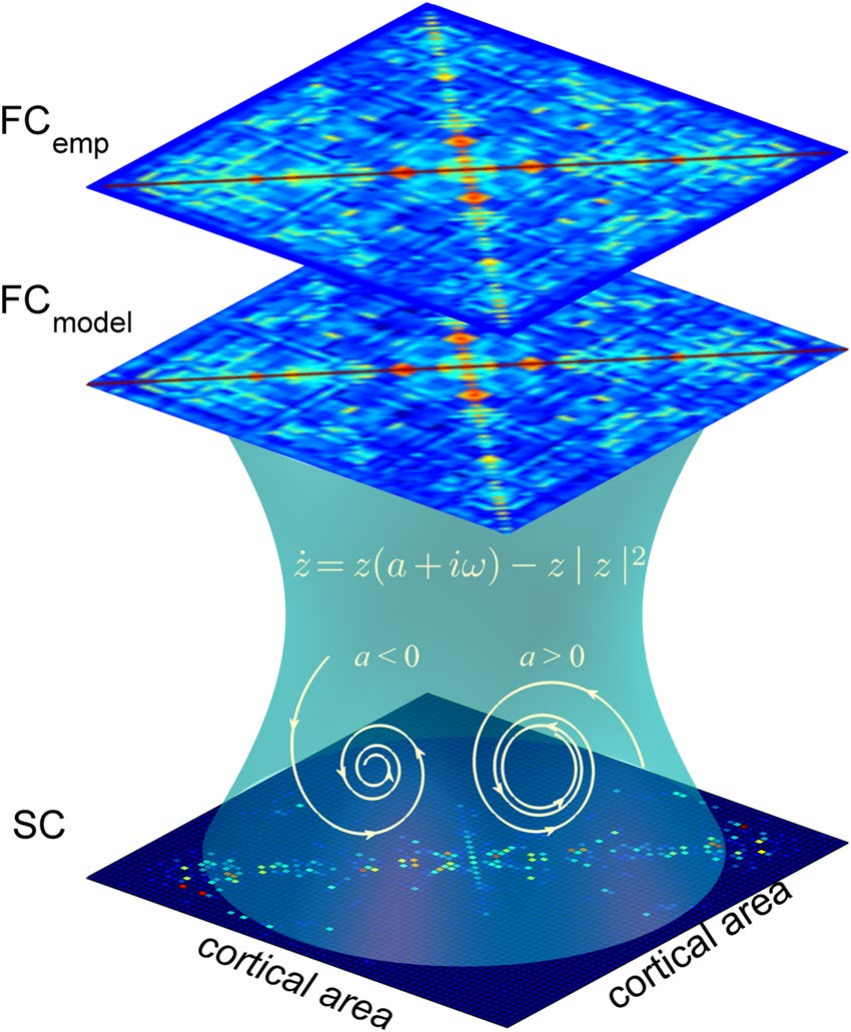
Whole-brain model linking anatomical connections and FC. The anatomical connectivity data were obtained using DTI averaged over a group of healthy participants. Using the AAL 90 parcellation we obtained a structural connectivity (SC) matrix linking 90 cortical and subcortical nodes with each other anatomically. Based on this matrix, a Hopf whole-brain computational model is built which simulates the resting activity of the 90 coupled brain areas. The simulated functional connectivity matrix (FC_model_) is then fitted to the empirical functional connectivity matrix (FC_emp_) for different model parameter combinations using the Euclidean distance between the values of FC_model_ and FC_emp_ (see *Methods*). With this framework the model parameter space can be explored in order to find the optimal parameter combination for each brain state.

**Figure 3:**
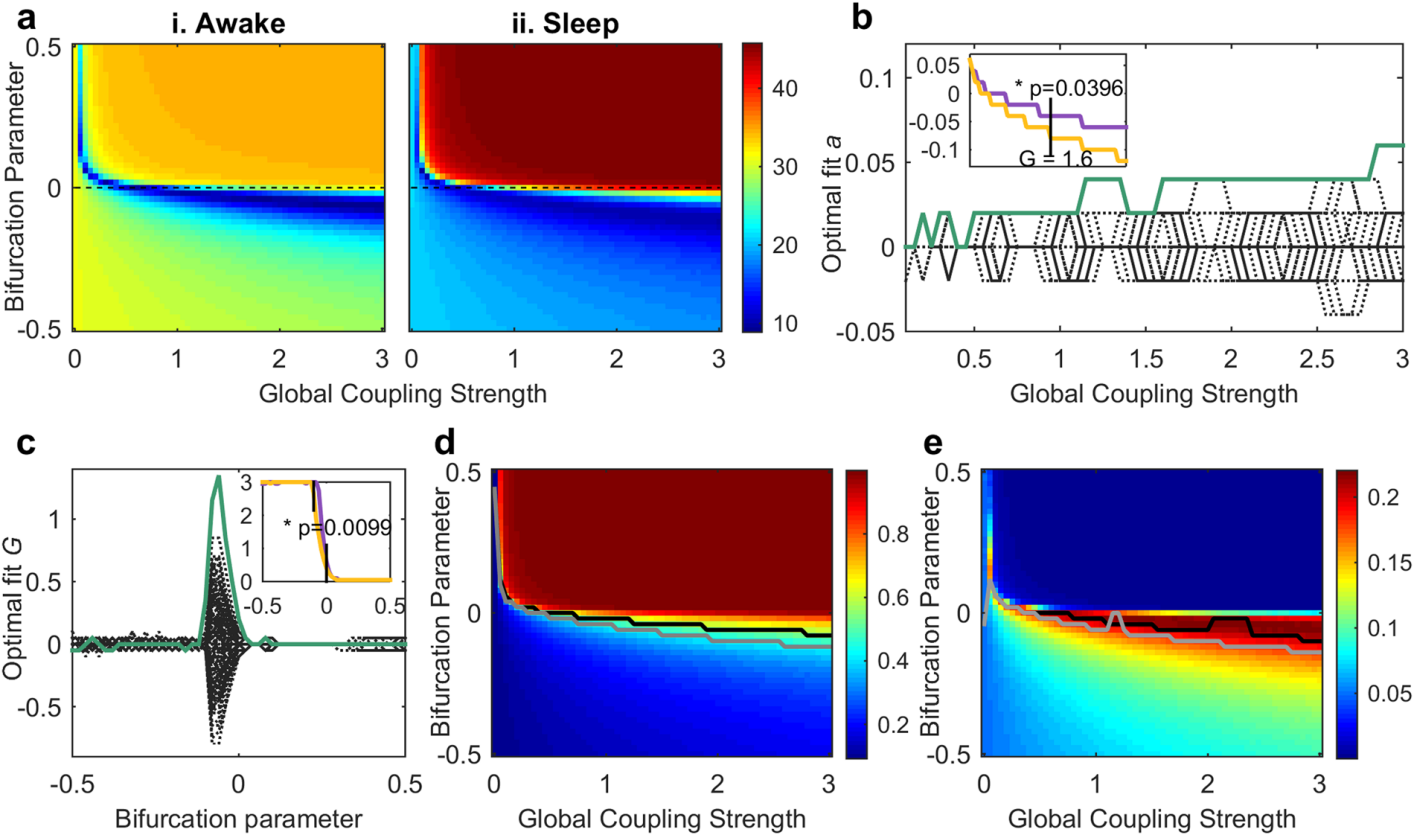
Whole-brain model parameter space exploration and fitting. (**a**) (i.,ii.) Euclidean distance between FC_model_ and FC_emp_ for different values of the global coupling strength G and the bifurcation parameter *a* in awake (i.) and sleep (ii.). Note that *a* is changed homogeneously over nodes. The optimal fit corresponds to a minimal Euclidean distance. In (**b**) the difference between the optimal Euclidean distance fit in awake and in sleep is shown as a function of *G*. The optimal fit is defined as the bifurcation parameter which corresponds to the minimum Euclidean distance for each value of *G*. The broken black lines represent the surrogate data constructed under the null-hypothesis of no difference between vigilance states, whereas the green line shows the difference between the optimal fit in awake and sleep. In the upper left corner the optimal fit for awake (violet) and sleep (yellow) is shown as a function of *G*. There is a significant difference between the two states from *G* = 1.6 onward with a corresponding p-value of 0.0396. In (**c**) the difference between the optimal Euclidean distance fit in awake and in sleep is displayed as a function of *a*. The broken black lines and the green line are to be read as in (**b**). In the upper right corner the optimal fit for awake (violet) and sleep (yellow) is shown as a function of *a*. There is a significant difference between the two states for −0.1 ≤ *a* ≤ 0 with a corresponding p-value of 0.0099. (**d**) represents the simulated global synchrony as a function of *G* and the bifurcation parameter *a*. The black line shows the optimal fit for the awake state, namely the closest value to the empirical global synchrony for each *G*, as a function of the global coupling strength. The gray line shows the same for the sleep state. In (**e**) the simulated metastability is displayed, also here as a function of *G* and the bifurcation parameter. The black and the gray line are to be read as in (**d**). Note the observable global shift to more negative bifurcation parameter values in (**d**) and (**e**).

To gain theoretical insights, we fitted a large-scale model of coupled nonlinear oscillators to the data of each of the vigilance states (see *Methods*). We compared the two vigilance states with regards to their dynamic working point, meaning the parameter region where the model fits the data best. We simulated the fMRI BOLD activity in each of the 90 brain regions by using the Hopf whole-brain model with the brain nodes coupled through the empirical structural connectivity (SC) matrix (see *Methods*+ Fig. 2). We performed a parameter space exploration by varying the two free parameters of the model: the global coupling parameter *G* and the bifurcation parameter *a*, where *a* was changed homogeneously over all nodes. We calculated the model’s FC matrix, phase synchrony and metastability for each parameter combination in the same fashion as for the empirical data. Then, we computed the best fit between the empirical and the simulated FC matrices for the two vigilance states (see *Methods*) and found a significant difference between the optimal bifurcation parameters in awake and sleep from a global coupling strength of 1.6 onwards (p-value 0.0396). In detail the optimal fit between empirical and simulated data lied in awake in the bifurcation parameter range between −0.04 and −0.06 and in sleep between −0.08 and −0.12. In general, we observed a better fit in the whole negative bifurcation parameter region in sleep as compared to awake from *G* = 0.4 onwards for awake and from *G* = 0.3 onwards for sleep (Fig. 3a,b). Regarding the global coupling parameter, we found that during wakefulness the optimal fit as a function of the bifurcation parameter lied in a higher range of *G*-values in the vicinity of the bifurcation (−0.1 ≤ *a* ≤ 0) than during sleep. This means that the connectivity strength between different brain areas was higher during wakefulness as compared to sleep (Fig. 3c).

Furthermore we analysed the optimal concordance in the parameter space between the simulated and empirical phase synchrony and equally for the metastability, and also here we found a shift of the optimal fit in sleep as compared to awake to more negative bifurcation parameter values and to lower global coupling strengths (Fig. 3d,e).

In summary, the dynamic working point shifted significantly to more negative bifurcation parameter values during sleep as compared to the awake state, where the system remained closer to the bifurcation. Additionally the system presented higher connectivity during wakefulness than during sleep, which benefits the propagation of interaction between different brain regions.

### Lowering of the effective connectivity during sleep

**Figure 4:**
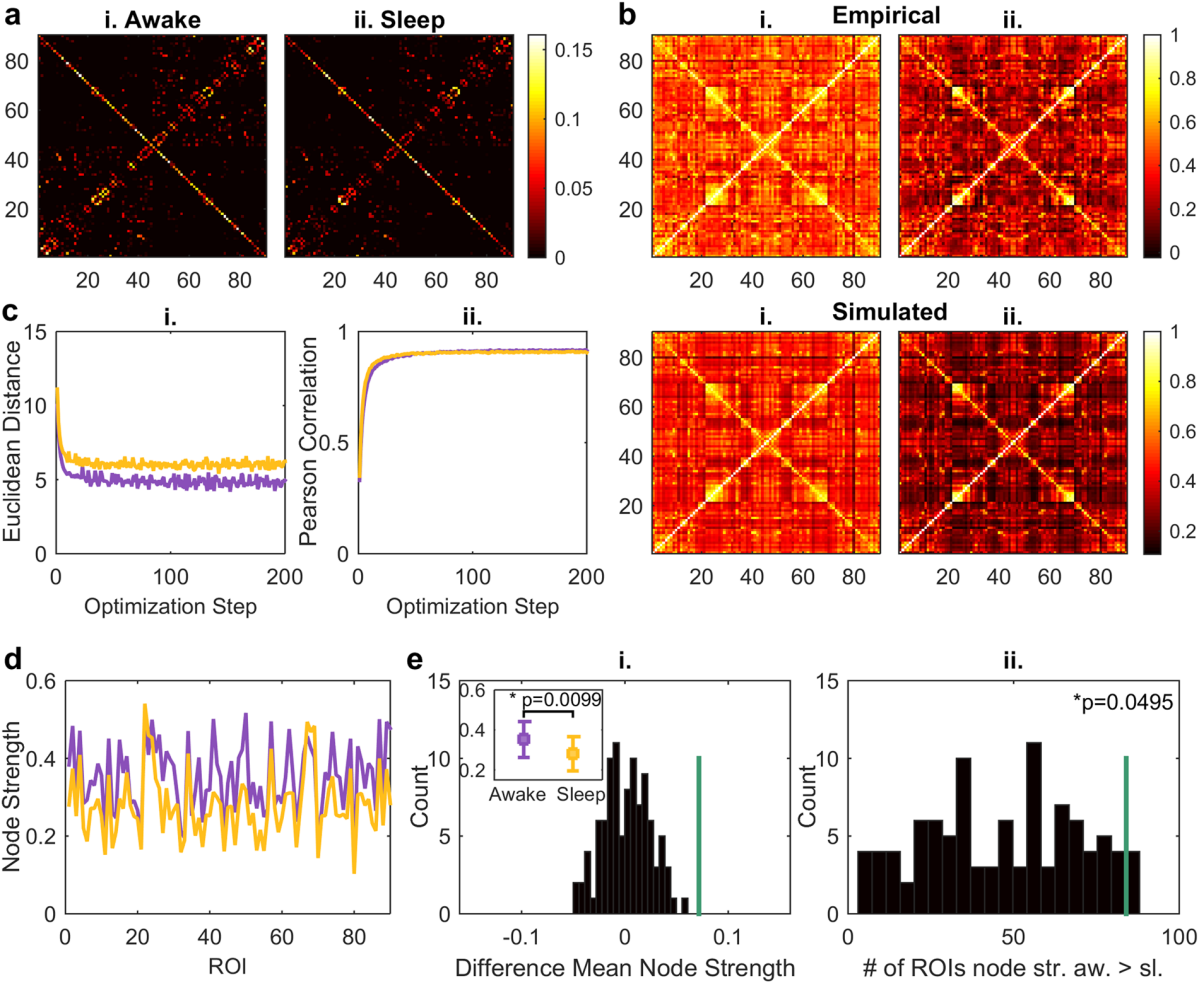
Effective connectivity in awake and sleep. In (**a**) the final effective connectivity (EC) matrices are shown in awake (i.) and in sleep (ii.). The above row in (**b**) displays the empirical FC matrices in awake (i.) and in sleep (ii.), whereas in the row underneath the simulated FC matrices ((i.) awake, (ii.) sleep) after the EC optimisation procedure are shown. (**c**) demonstrates the fitting as a function of the iteration steps: in (i.) the Euclidean distances between FC_model_ and FC_emp_ for awake (violet) and sleep (yellow) are shown, and in (ii.) the Pearson correlation coefficients between the two matrices are represented (colour code as in (i.)). In order to assess the differences between the EC matrices in both modalities and to investigate whether the differences are of global or local nature, in (**d**) the node strength of the EC matrices are displayed (colour code as in (**c**)). With this, node-wise EC values are obtained. (**e**)(i.) shows the difference between the mean node strength in the awake and the sleep state. The histogram (black) represents the distribution of the test-statistic under the null-hypothesis of no difference between vigilance states, whereas the green line shows the difference of the mean node strengths of the actual EC matrices. In the upper left corner the mean node strengths and their standard deviations are shown. They are significantly different with a p-value of 0.0099. In (ii.) the number of nodes with node strength higher during wakefulness than during sleep are displayed. As before, the histogram represents the test-statistic and the green line the actual number of nodes. The number of nodes with higher node strength in awake than in sleep are significantly higher than obtained with the state-shuffling surrogate data (p-value: 0.0459). Note that the number of ROIs exhibiting higher EC in awake than in sleep is 84 out of 90 possible nodes, suggesting that the difference is of global nature.

In order to determine the actual level of interaction and connectivity between different brain areas, we investigated the differences between the two brain states by studying the changes in the effective connections between different brain nodes. Here we define effective connectivity (EC) as a combination of anatomical connectivity and connection weights (i.e., synaptic conductivity), meaning the effective interaction between different brain areas. The EC was estimated by iteratively updating the weights of the non-zero anatomical connections with the weighted difference between the empirical and the simulated FC matrix (see *Methods*). In each iteration step the Euclidean distance between the empirical and the simulated FC matrix was calculated and if this distance was smaller than the previous one, the EC matrix was updated with the optimised structural weights (see *Methods*). This optimisation procedure was performed for 200 iterations, after which the fitting has already reached a stable value (Fig. 4c i.,ii.).

Comparing the resulting final EC matrices between the two brain states, we observed a global lowering of the strength of the effective connections in sleep with respect to the awake state (Fig. 4a i.,ii.). In order to assess these differences we computed the node strength of the EC matrix for each of the 90 brain nodes (Fig. 4d). We found that the mean node strength of the effective connections was significantly higher in awake than in sleep (awake: 0.3520 ± 0.0898, sleep: 0.2804 ± 0.0854, p-value: 0.0099) (Fig. 4e i.). In addition to that we investigated the local differences between effective connection node strengths and we found that the total number of nodes displaying higher node strength during wakefulness with respect to sleep was significantly higher in the data as compared to the surrogate data. (84 nodes, p-value 0.0495) (Fig. 4e ii.).The few regions displaying higher node strength during sleep were the calcarine sulcus (left and right), the cuneus (left and right), the lingual gyrus (right) and the paracentral lobule (left). The 10 regions with the highest difference in node strength between wakefulness and sleep were in order from the highest difference onward:

Superior temporal gyrus (right); Precentral gyrus (right); Superior temporal gyrus (left); Postcentral gyrus (right); Putamen (left); Postcentral gyrus (left); Insula (left); Inferior frontal gyrus, Pars opercularis (right), Middle occipital gyrus (left) and Inferior occipital cortex (left).

Overall, the effective connections were significantly lower during sleep than during wakefulness. Being this the case for the mean over nodes and also for 93% of the local brain nodes, we suggest, that this observable difference is of global rather than local nature.

### Validation of the effective connectivity modelling procedure

Next, we validated the procedure for computing the effective connectivity by applying a simple model validation simulation. We computed the EC matrix starting from the optimal simulated FC matrix, obtained through the original EC calculation (Fig. 4 simulated b i.,ii.), instead of using the empirical one (Fig. 4 empirical b i.,ii.) (see *Methods*). We correlated the resulting validation EC matrix with the original one and found a Pearson correlation coefficient of 0.992, meaning that the procedure for computing the EC matrix was reliable.

### Theoretical model response to external stimulus

**Figure 5:**
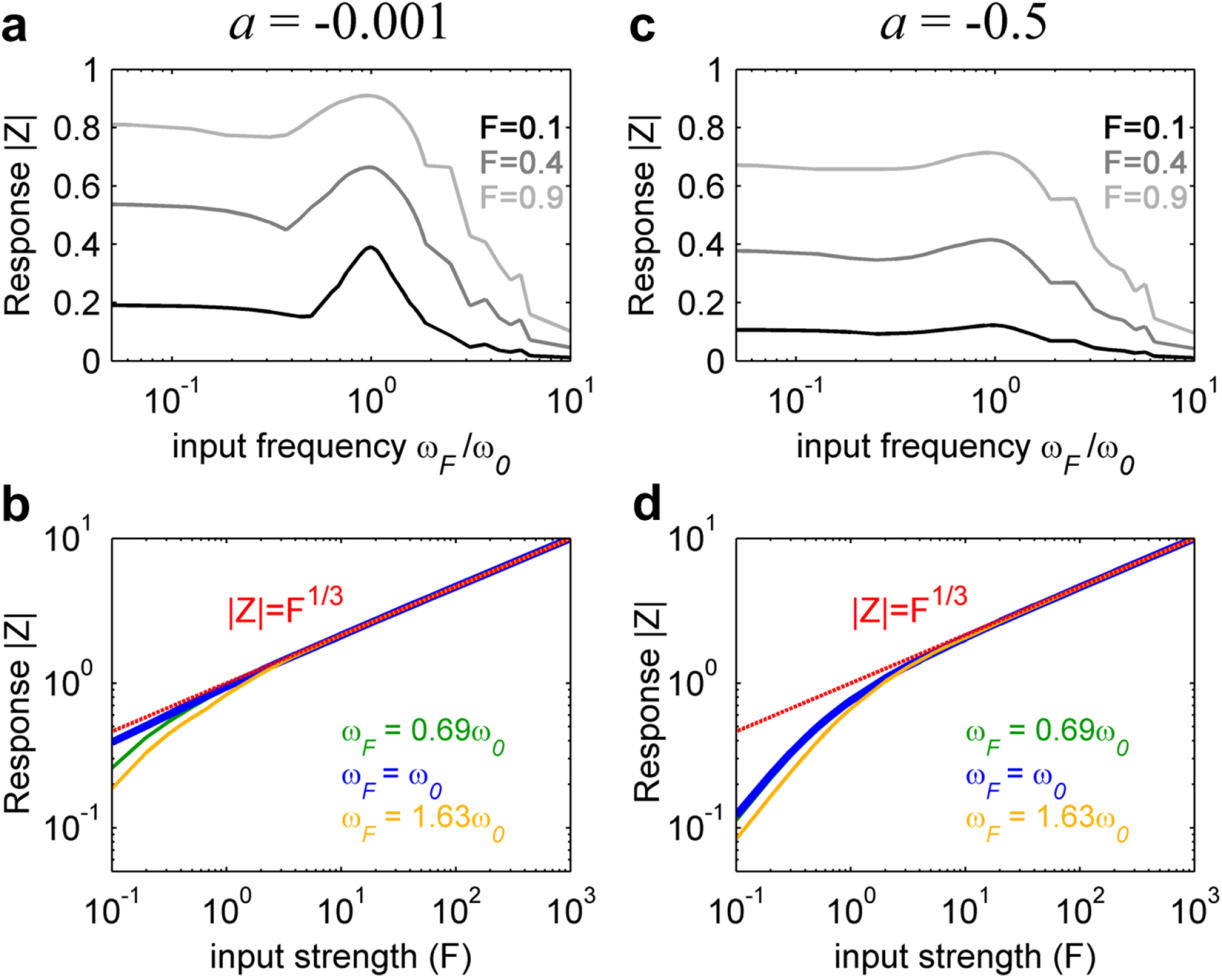
Single node response to external stimulus for two different dynamic working regimes. In (**a**) the response – the absolute value of the simulated complex signal |Z| – of a single node is shown as a function of the input frequency for different input strengths *F*. *ω*_0_ is the intrinsic frequency, which was here set to 1. (**b**) displays the model response as a function of the input strength for different input frequencies. The red line shows hypothetical power law behaviour. Both (**a**) and (**b**) are simulated with the bifurcation parameter *a* very close to the bifurcation at *a* = 0. (**c**) and (**d**) show the same as (**a**) and (**b**), with the difference that the bifurcation parameter used for the simulations was set to a negative value in the noisy regime of the model. Note that for *a* close to the bifurcation the model response follows the power law $Z\propto {F}^{\frac{1}{3}}$ for an input frequency equal to *ω*_0_, whereas for *a* in the negative regime this is not the case: weak inputs are no more amplified. In this analysis no noise was added to the system.

We have shown that the awake state is associated with a model in which the whole system is positioned closer to a Hopf bifurcation, meaning *a* closer to 0. This is a functionally relevant feature, since it is known that, close to the bifurcation, the Hopf model has optimal resonance and nonlinear amplification behaviours^27^. Indeed, if the system is subjected to a sinusoidal stimulus $F(t)=F{e}^{i{\omega }_{F}t}$ at frequency *ω*_*F*_, the node’s response presents a resonance when it is stimulated at its intrinsic frequency *ω*_*F*_ = *ω*_0_ (Fig. 5a,b). Moreover, in the vicinity of the bifurcation and if the node is stimulated at its intrinsic frequency, the amplitude of the response, |*Z*|, follows the power law $Z\propto {F}^{\frac{1}{3}}$ (Fig. 5c,d). Such power law relation indicates that weak inputs are highly amplified, while strong inputs elicit responses with lower gain. This makes the network to have a large dynamic range of responsiveness. In conclusion, in the vicinity of the bifurcation the node presents sharp frequency selectivity with high sensitivity allowing for a better communication between nodes and a better reaction to external stimuli.

## Discussion

We used data- and model-driven analyses to compare the brain states of two different behavioural conditions, namely wakefulness and slow-wave sleep. Using the whole-brain model we found a significant shift of the brain’s global working point from the edge of the transition between noisy and sustained oscillatory behaviour during wakefulness to a noisy regime characterised by a stable focus during slow-wave sleep as a possible mechanistic explanation of the observed empirical functional changes between those two brain states. We also found that the effective connectivity is reduced in slow-wave sleep compared to wakefulness. Overall, this suggests that the propagation of external perturbations is decreased in slow-wave sleep compared to wakefulness, with incoming inputs being transmitted with sharper selectivity and higher sensitivity during wakefulness.

Interestingly, in our data-driven analysis, we found that the metastability (i.e. the standard deviation of the phase synchrony) decreases during sleep (Fig. 1d). This is particularly interesting given the recent focus on the non-stationarity of resting-state activity, where studies have demonstrated that functional correlations are dynamic and evolve over time^28–32^. Importantly, Ponce-Alvarez and colleagues have shown that the global phase synchrony of the BOLD signals evolves at a very slow time scale of <0.01 Hz and that with this variability the system visits different synchronised brain states^33^. If, as observed in the case of slow-wave sleep, this variability decreases compared to wakefulness, the dynamical repertoire of the brain during this vigilance state could be limited, indicating that the brain must operate in a different dynamic working region. The finding that slow-wave sleep is more constrained by the underlying structural connectivity further supports this interpretation^21^.

This fits with our further demonstration that, on average, the functional connectivity is lower during sleep as compared to wakeful rest (Fig. 1a,b). This finding is consistent with previous studies showing that the resting-state networks are generally preserved during sleep, but with altered connectivity strength^12, 14^. These previous studies show that in slow-wave sleep the cortico-cortical functional connections lose their strength^12, 14–16^, in particular, almost all cortico-cortical connections in the AAL template are reduced during deep sleep^18, 34^. This clearly demonstrates that the functional connectivity decreases on a global level.

Furthermore, we have shown that the mean phase synchronisation is lower during sleep than during wakefulness (Fig. 1c). This result supports the fact that the communication in the brain is constrained during slow-wave sleep, as has been found in intracerebral (EEG) recordings, where slow oscillations appear often out-of-phase in different brain regions and appear as events of a local nature^35^. While these results are valid for electrophysiological data, it has been demonstrated that slow cortical potentials recorded with intracerebral EEG show a similar correlation structure as spontaneous BOLD fluctuations during slow-wave sleep^36, 37^.

In order to find a mechanistic explanation of the empirical results and to learn more about the underlying dynamics, we applied a whole-brain model based on the normal form of a supercritical Hopf bifurcation. We showed that the region where the model fits the data best lies during wakefulness close to the bifurcation, on the edge between noise-induced and self-sustained oscillations, whereas during sleep it shifts to a more negative regime characterised by noisy oscillations. Furthermore, we demonstrated that during wakefulness the optimal global coupling parameter value as a function of the bifurcation parameter is higher in the vicinity of the bifurcation than during sleep (Fig. 3). These results indicate that the brain is operating in a different dynamical regime during deep sleep when compared to wakeful rest. The brain’s global working point presents higher connectivity and less stability during wakefulness, suggesting that the propagation of activity is increased in this brain state. This allows for better communication between different brain areas and an improved reaction to stimuli during wakefulness. Increased stability implies a failure to amplify weak stimuli, as required, for instance, for the firing of few cells in V1 to globally propagate to the fronto-parietal cortex during conscious access of visual information, according to the Global Neuronal Workspace theory proposed by Dehaene and Changeux^38–40^. This ignition of the global workspace is facilitated during wakefulness, instead, since dynamics are posed near the bifurcation point (Fig. 5). Our analysis was global in nature and thus could not localise the dynamical changes to sensory areas or the fronto-parietal cortex. Further analyses are needed to identify if the dynamical changes are due to global processes or in fact to local changes influencing the system on a global level. A possible way of expanding the current method in order to look into local region wise changes could be to adapt the bifurcation parameter for each node instead of taking one value homogeneously for all the regions as is done here. In fact this approach has been performed by Deco *et al*.^26^, where they optimised the spectral characteristics of each local brain region and thus gained a heterogeneous optimal working point for each brain region. Nevertheless, the here presented method shows a clear shift in the bifurcation parameter and in the global coupling strength, which is an indication for this phenomenon to be more of global nature than of a local one. This is supported by the fact, that for a fixed bifurcation parameter *a* the model for sleep becomes the model for wakefulness when *G* = *G* + Δ*G*, as can be observed in Fig. 3a. The whole system is shifted between the two brain states. By increasing the global coupling strength, the mean node strength of the SC matrix is increased, which directly influences the simulated FC matrices in a global way, resulting in a better fit with the empirical FC matrix during wakefulness. On the contrary, decreasing the global coupling strength results in a better fit with the empirical FC matrix during sleep. We tested the scenario of equal FC node strengths in wakefulness and sleep and found that indeed the differences in the dynamical range which fits the data best vanish (see Supplementary Fig. S2). Still, we cannot exclude that local region wise changes are responsible for the globally observed effects.

Another important point is the fact that the Hopf model possesses optimal resonance and amplification behaviour when close to the bifurcation as shown in Fig. 5. We showed that in the vicinity of the bifurcation, as it is the case during wakefulness, the system demonstrates a large dynamic range of responsiveness, showing frequency specificity and power-law behaviour. The proximity of the optimal dynamical working point to the bifurcation during wakefulness is functionally relevant in the sense that in this regime sensory inputs are transmitted with sharper selectivity and higher sensitivity due to a better communication between nodes. Furthermore the network has the largest dynamic range of responsiveness. These properties are important and characteristic for an awake state. Additionally the response to stimuli is non-linear. For lower bifurcation parameters, as during deep sleep, we do not observe these properties, and instead the response is dominated by the linear terms^27^. This implies further that close to the bifurcation the system needs to have a higher level of complexity to show the previously mentioned characteristics. This confirms the findings in the studies performed by Massimini and colleagues^23, 25, 41, 42^, where the level of consciousness is assessed by measuring the Perturbative Complexity Index (PCI), characterizing the deterministic cortical responses to external perturbations. It has been observed that the PCI is lower during an unconscious state such as slow-wave sleep. This agrees with the fact that during sleep the system is located in the negative bifurcation parameter regime, where it is less complex in the sense that the dynamic range of responsiveness is smaller.

Besides the parameter space exploration of the model, we also simulated the effective connections between different brain areas in order to get a better insight into the actual level of connectivity between different brain nodes. We found decreased effective connectivity on a global level during slow-wave sleep in comparison to wakefulness (Fig. 4). Effective connectivity is usually defined as a causal connectivity measure, meaning the directional influences of one brain area or neural element over another^23, 43–45^. In this work we have applied another approach: here the effective connections are estimated using the anatomical connections, the functional connections and the dynamics given by the model. They can be interpreted as the synaptic weights between the different brain nodes, which are not captured by the structural or functional connections alone. Effective connectivity can be understood as the biophysical “mechanistic causes” of the apparent changes in the functional connections, given that we can explain those changes with changed effective interactions in only existing anatomical links given by the anatomical connections. The observable decrease in EC during deep sleep indicates a drop of integration in the brain on the global level: the communication between different brain areas is limited. This result is compatible with the Integrated Information Theory of Consciousness, which states that consciousness corresponds to the capacity of the brain to integrate information, and other studies which have shown that integration is impaired during unconsciousness^40, 46^. Furthermore it is evident, when looking at the node strength for each brain area (Fig. 4d), that the effective connections in the two brain states are only a scaled version of one another. A possible interpretation of this particular result could be that during wakefulness the system demonstrates higher excitability which supports the fact that excitability increases with the time awake^47, 48^. When looking more deeply into the set of regions which show the highest differences in node strength between wakefulness and sleep, we observed that most of these regions are related to the processing of sensory information (superior temporal: auditory, somatosensory: postcentral), motor planning (precentral gyrus which contains the primary motor cortex) and include a subcortical structure (putamen), which is densely connected to both primary somatosensory and motor cortices. The functional isolation of these areas from the rest of the cerebral cortex could indicate diminished levels of arousal during deep sleep. Such diminished arousal comprises lack of motor activity and increased thresholds for awakening - with awakenings due to stimulation during sleep being generally related to either somatosensory and/or auditory stimulation.

The results from the dynamical brain model described in this article were obtained using fMRI BOLD signals, but similar, matching brain activity patterns are shown in EEG signals. Deep sleep is characterised by the onset of high amplitude delta (1–4 Hz) waves, which indicates an increment in the local synchronisation of neural populations^49^. Deep sleep, such as other states of unconsciousness, is governed by bistable oscillations that reflect alternating periods of firing and neural quiescence. This stereotypical pattern of neural firing entails a decrease in the differentiation of brain activity which is related (via the information-integration theory) to diminished conscious awareness^50^.

Our results obtained from modelling whole-brain BOLD dynamics and connectivity reflect these and other electrophysiological observations. Increased stability of oscillations has been reported for the unconscious state induced by propofol, a general anaesthetic drug^51, 52^. Furthermore, the effective connectivity of neural activity measured using EEG is notoriously reduced during unconscious states such as deep sleep, general anaesthesia and in patients with disorders of consciousness. While in many of these brain states EEG recordings yield patterns of activity with seemingly high levels of local and global synchronisation, the propagation of externally induced perturbations (using transcranial magnetic stimulation [TMS]) is damped during unconscious brain states^25^. We can speculate that the loss of stability, global coupling and effective connectivity revealed from fMRI data using our computational model represents the hemodynamic counterpart to these results.

This speculation is consistent with combined EEG-fMRI and PET studies that relate fluctuations in brain metabolism to delta band power during deep sleep. The work of Dang-Vu *et al*.^53^ established that frontal metabolism presents an inverse correlation with delta band oscillations during deep sleep. A similar result was obtained by Lei *et al*.^54^ using concurrent EEG-fMRI recordings. In this paper, Lei *et al*. showed that fronto-parietal BOLD signals present an inverse correlation with fluctuations in delta power during deep sleep. These studies suggest that frontal (and possible parietal) BOLD signals reflect fluctuations in delta power and that their computational modelling might, in turn, yield insights about the stability and effective connectivity of slow oscillations during deep sleep. To which extent BOLD signal fluctuations from other brain areas reflect slow activity or other independent electrophysiological phenomena remains to be investigated in the future.

To summarise, in this work we have suggested a possible mechanistic explanation of the empirical functional changes observed during slow-wave sleep. We have shown that the dynamic working point of the human brain is significantly different during slow-wave sleep compared to wakeful rest. We have demonstrated that during deep sleep the system shifts to a noisy oscillatory state, whereas during awake it stays closer to the bifurcation, and discussed how this might allow the brain to better process information in more complex ways during wakefulness. We have also shown that the brain’s global working point presents higher connectivity during wakefulness than during sleep. We suggest that these dynamical changes occur mainly on a global rather than a local scale. This claim is further supported by the fact that the effective connections between different brain areas decrease globally during sleep, which suggests a higher level of integration and excitability during wakefulness on a whole-brain level. Further studies are required to verify if these changes are in fact due to effects on a global level or if the observable effects can be explained by a group of local nodes driving the dynamic system.

Overall, by exploring the mechanistic properties of whole-brain dynamics in two different behavioural states, wakefulness and slow-wave sleep, we have added complementary evidence to the developing understanding of the brain as a complex system that supports widely different purposes. Importantly the whole-brain modelling allowed us to explore features of the functional connectome not immediately tractable to standard analysis strategies.

## Materials and Methods

### Experimental data

#### Participants

In this study we included a total of 18 young, healthy consecutive participants with data of sufficient quality Written informed consent was obtained from all subjects. The experimental protocol was approved by the local ethics committee “Ethik-Kommission des Fachbereichs Medizin der Goethe-Universität Frankfurt am Main, Germany” with the ethics application title “Visualisierung von Gehirnzuständen in Schlaf und Wachheit zum Verständnis der Abnormitäten bei Epilepsie und Narkolepsie” and the assigned number: 305/07. The subjects were reimbursed for their participation. The applied methods were carried out in accordance with the relevant guidelines and regulations.

Participants entered the scanner in the evening and underwent a resting state fMRI session with simultaneous EEG acquisition lasting for 52 minutes. Participants were not instructed to fall asleep, but were asked to relax, close their eyes and not to fight sleep. Lights were dimmed in the scanner room and subjects were shielded from scanner noise using earplugs. For the day of the study all participants reported a wake up time between 5:00 AM and 11:00 AM the night before and a sleep onset time between 10:00 PM and 2:00 AM. These values remained similar throughout the 6 days prior to the experiment.

All of the 18 participants included in this study (8 females, mean ± SD age of 23.1 ± 2.6 years) reached deep sleep (N3) as determined by sleep staging simultaneously acquired polysomnography data according to the standard rules of the American Academy of Sleep Medicine^2^. For these participants the mean (±SD) durations of contiguous sleep epochs were 12.37 ± 6.61 minutes for wakefulness, 8.52 ± 2.83 minutes for N1, 14.69 ± 5.72 minutes for N2 and 16.56 ± 8.39 minutes for N3. These subjects were part of a larger cohort (63 participants in total, 36 females, mean ± SD age of 23.4 ± 3.3 years). A sub-selection of participants was necessary in order to obtain comparable stretches of time in each sleep stage, since only the aforementioned 18 subjects reached N3 sleep. In this study only wakefulness and deep sleep (N3) were considered.

#### fMRI and EEG data collection

EEG via a cap (modified BrainCapMR, Easycap, Herrsching, Germany) was recorded continuously during fMRI acquisition (1505 volumes of T2*-weighted echo planar images, TR/TE = 2080 ms/30 ms, matrix 64 × 64, voxel size 3 × 2 × 2 mm^3^, distance factor 50%; FOV 192 mm^2^) at a 3 T Siemens Trio (Erlangen, Germany). An optimised polysomnographic setting was employed (chin and tibial EMG, ECG, EOG recorded bipolarly [sampling rate 5 kHz, low pass filter 1 kHz] with 30 EEG channels recorded with FCz as the reference [sampling rate 5 kHz, low pass filter 250 Hz]. Pulse oxymetry and respiration were recorded via sensors from the Trio [sampling rate 50 Hz]) and MR scanner compatible devices (BrainAmp MR+, BrainAmpExG; Brain Products, Gilching, Germany), facilitating sleep scoring during fMRI acquisition^2^.

MRI and pulse artefact correction were performed based on the average artefact subtraction (AAS) method^55^ as implemented in Vision Analyzer2 (Brain Products, Germany) followed by objective (CBC parameters, Vision Analyzer) ICA-based rejection of residual artefact-laden components after AAS resulting in EEG with a sampling rate of 250 Hz. EEG artefacts due to motion were detected and eliminated using an ICA procedure implemented in Vision Analyzer2. Sleep stages were scored manually by an expert according to the AASM criteria^2^. This type of data has been published and well described in several publications^11, 16, 56^.

#### fMRI pre-processing

Using Statistical Parametric Mapping (SPM8, www.fil.ion.ucl.ac.uk/spm) Echo Planar Imaging (EPI) data were realigned, normalised (MNI space) and spatially smoothed (Gaussian kernel, 8 mm3 full width at half maximum). Data was re-sampled to 4 × 4 × 4 mm resolution to facilitate removal of noise and motion regressors. Note that re-sampling introduces averaging of Blood Oxygen Level Dependent (BOLD) signals, which are finally averaged over cortical and sub-cortical regions of interest (determined by the automatic anatomic labelling [AAL] atlas). Cardiac, respiratory (both estimated using the RETROICOR method^57^) and motion-induced noise (three rigid body rotations and translations, as well as their first 3 temporal derivatives, resulting in 24 motion regressors) were regressed out using least squares and retaining the residuals. Data was band-pass filtered in the range 0.01–0.1 Hz^58^ using a sixth order Butterworth filter.

To further control for possible differences in head motion in the different brain states, which could, even if regressed out, still influence the outcome of the data analyses, we computed the framewise displacement for both vigilance states and compared the outcome between the states. To compute the framewise displacement we followed the procedure described in Power *et al*.^59^. There were no significant differences using a Wilcoxon rank test (awake: 0.1521 ± 0.0095 mm, sleep: 0.1406 ± 0.0110 mm; p = 0.419 (mean ± SEM)) (see Supplementary Fig. S3).

### DTI data collection and processing

We used the normal structural connectome obtained using DTI in 16 healthy right-handed participants (11 men and 5 women, mean age: 24.75 ± 2.54), recruited through the online recruitment system at Aarhus University. In this study, participants with psychiatric or neurological disorders (or a history thereof) were excluded from participation. The MRI data (structural MRI, DTI) were recorded in a single session on a 3 T Siemens Skyra scanner at CFIN, Aarhus University, Denmark. The following parameters have been applied for the structural MRI T1 scan: voxel size of 1 mm^3^; reconstructed matrix size 256 × 256; echo time (TE) of 3.8 ms and repetition time (TR) of 2300 ms.

The DTI data were collected using TR = 9000 ms, TE = 84 ms, flip angle = 90°, reconstructed matrix size of 106 × 106, voxel size of 1.98 × 1.98 mm with slice thickness of 2 mm and a bandwidth of 1745 Hz/Px. Furthermore, the data were recorded with 62 optimal nonlinear diffusion gradient directions at *b* = 1500 s/mm^2^. One non-diffusion weighted image (*b* = 0) per 10 diffusion-weighted images was acquired, approximately. Additionally, the DTI images were recorded with different phase encoding directions. One set was collected applying anterior to posterior phase encoding direction and the second one was acquired in the opposite direction. We used the automated anatomical labelling (AAL) template to parcellate the entire brain into 90 regions (76 cortical regions and 14 subcortical regions, AAL90). The parcellation contained 45 regions in each hemisphere^53^. In order to co-register the EPI image to the T1-weighted structural image, we used the linear registration tool from the FSL toolbox (www.fmrib.ox.ac.uk/fsl, FMRIB, Oxford)^60^. We co-registered the T1-weighted image to the T1 template of ICBM152 in MNI space. The resulting transformations were concatenated and inversed and further applied to warp the AAL template^61^ from MNI space to the EPI native space, where we preserved the discrete labelling values by applying interpolation using nearest-neighbour method. Accordingly the brain parcellations were conducted in each individual’s native space. The acquired DTI data was used to generate the structural connectivity (SC) maps for each participant. The two recorded datasets were processed, each with different phase encoding to optimise signal in difficult regions. To construct these structural connectivity maps we applied a three-step process. First, we defined the regions of the whole-brain network with the AAL template as used in the functional MRI data. Secondly, we used probabilistic tractography to estimate the connections between nodes in the whole-brain network (i.e. edges). Finally the data was averaged across participants.

In order to ensure that the resulting group SC matrix was representative of the average single subject and that no unwanted biases were introduced by averaging across subjects, we show the single subject SC matrices in Supplementary Fig. S4. Furthermore we performed a consistency analysis following Roberts *et al*.^62^ (see Supplementary Fig. S4).

In accordance with the procedure applied for analysing the rs-fMRI data, the AAL template was used to parcellate the entire brain into AAL90. In order to co-register the b0 image in diffusion MRI space to the T1-weighted structural image and then to the T1 template of ICBM152 in MNI space^63^, we used the FLIRT tool from the FSL toolbox (www.fmrib.ox.ac.uk/fsl, FMRIB, Oxford). We concatenated and inversed the two transformation matrices from the described co-registration steps and applied them correspondingly to warp the AAL templates^61^ from MNI space to the diffusion MRI native space.

### Construction of surrogate data

In order to assess the statistical significance of the results found in this study, surrogate data were constructed under the null-hypothesis of no difference between conditions (wakefulness and slow-wave sleep) based on randomly shuffled group assignments. Each of the 18 datasets consists of an awake session and a sleep session recorded in the same subject. Based on these original data sets, surrogate data were created by randomly shuffling the vigilance state assignments with a probability of 0.5. This means that the group sizes remained the same as in the original data, with the difference that for each subject there existed a 50% chance that its brain state assignments were switched between the two recordings. In this way the recordings were not mixed between participants, but the vigilance conditions were randomly shuffled within each group instead. Applying this method, there exists a 50% chance that a data pair “awake-sleep” either remains “awake-sleep” or becomes “sleep-awake”, meaning that the groups get randomly mixed and thus fulfilling the null-hypothesis of no difference between conditions. Due to computational demands 100 surrogate datasets were produced which resulted in a minimum possible p-value of 0.0099.

### Group averaged functional connectivity matrices

First, the signals were detrended and demeaned before they were band-pass filtered within the range of 0.04–0.07 Hz following Glerean *et al*.^64^. In order to be able to extract the instantaneous phases of the BOLD signals (see ‘Phase synchrony and metastability’), the signals must be filtered within a narrow band. We chose the frequency range of 0.04–0.07 Hz because this frequency band has been mapped to the gray matter and it has been shown to contain more reliable and functionally relevant information compared to other frequency bands and to be less affected by noise^3, 64–66^. Subsequently the filtered time series were z-scored, meaning that the mean was subtracted and they were divided by their standard deviation. This was done for each subject, because the standard deviation of the BOLD signal is subject-specific. Next, we calculated the functional connectivity (FC) matrices for each of the 18 participants for each of the two recordings. The FC matrix is defined as the matrix of Pearson correlations between the BOLD signals of all pairs of regions of interest (ROIs) in the AAL atlas over the whole acquisition duration. Fixed-effect analysis was used to obtain group-level FC matrices, meaning that the Fisher’ r-to-z transform [*z* = tanh(*r*)] was applied to the correlation values before averaging over participants within the two vigilance states and back-transforming to correlation values. This resulted in two final FC matrices, one for each condition. In order to compare the averaged FC matrices between the two vigilance states, we calculated the mean and standard deviation of the upper triangle matrix of both of the FC matrices. We subtracted the resulting mean value for the sleep state from the mean value of the awake state. Finally, to test for significance, the same procedure was performed on the surrogate data sets.

### Brain state classification with Gaussian classifier

In order to establish how specific the functional connectivity is to the condition (i.e. wakefulness and slow-wave sleep), we classified the brain state based on the covariance of fMRI signals using a jackknife cross-validation approach, assuming that observations are drawn from a multivariate Gaussian distribution. First, for each vigilance state, the data of *n* − 1 participants (train set) was used to estimate the covariance (Σ_awake_ and Σ_sleep_), where *n* is the number of participants. Note that since the data was z-scored, the mean of each fMRI time-series was zero and, thus, in the Gaussian approximation, the fMRI signals were fully determined by their covariance. Second, the data of the remaining subject (test set) was associated to a vigilance state by choosing the zero-mean multivariate Gaussian process (*N*(0,Σ_awake_) or *N*(0,Σ_sleep_)) that maximises the log-likelihood of the test data given the trained model. The percentage of correct classifications was computed across the *n* participants. The likelihood of a test N-dimensional vector *X*_*t*_, representing the *t*-th time step of the test data, given the zero-mean multivariate Gaussian process *N*(0,Σ), is given by:

1 $$P({X}_{t}|{\rm{\Sigma }})={[2\pi \det ({\rm{\Sigma }})]}^{-\frac{1}{2}}\exp (-\frac{1}{2}{X}_{t}^{\ast }{{\rm{\Sigma }}}^{-1}{X}_{t}),$$

where $\det ({\rm{\Sigma }})$ is the determinant of the covariance Σ and the superscript * is the transpose. Assuming independence of the observations, the log-likelihood *L* of the entire test time series *X* = *X*_1,...,*T*_, where *T* is the number of time steps, is given by:

2 $$L(X|{\rm{\Sigma }})=\,\mathrm{log}\,{\prod }_{t=1}^{T}P({X}_{t}|0,{\rm{\Sigma }})={\sum }_{t=1}^{T}\mathrm{log}\,P({X}_{t}|{\rm{\Sigma }})$$

In summary, for each test data *X*, we calculated *L*(*X*|Σ_awake_) and *L*(*X*|Σ_sleep_) and if *L*(*X*|Σ_awake_) > *L*(*X*|Σ_sleep_), the predicted vigilance state was awake, otherwise the predicted vigilance state was sleep.

To assess statistical significance of the classification performance we calculated the probability of getting *k* correct classifications (hits) by chance, which is given by: $\Pr (k)={C}_{n}^{k}{p}^{k}{(1-p)}^{n-k}$, where *p* is the probability of getting a hit by chance $(p=\frac{1}{2})$ and *n* is the number of tests. Significant decoding was reached when the decoding performance exceeded the 95th percentile of $\Pr (k)$.

We used a similar procedure to evaluate how similar the time-series of a single subject are to a random sample from the grouped time-series of the other *n*-1 subjects, in each behavioural condition. Let *X* ^(*i*)^ be an N-by-T matrix containing the time-series of the i-th subject. We estimated the covariance Σ_train_ using the data from the remaining *n*-1 subjects, but excluding T N-dimensional vectors randomly selected that form a surrogate N-by-T time-series *X*_pseudo_. We then compared the ratio between the log-likelihoods *L*(*X* ^(*i*)^|Σ_train_) and *L*(*X*_pseudo_|Σ_train_), i.e., *r*_*i*_ = *L*(*X* ^(*i*)^|Σ_train_)/*L*(*X*_pseudo_|Σ_train_), using Equations 1–2. We repeated this procedure for 5,000 random selections of *X*_pseudo_ and calculated the average log-likelihood ratio 〈*r*_*i*_〉. We expected that 〈*r*_*i*_〉~1 if the time-series of the i-th subject were indistinguishable from a random sample taken from the time-series of the remaining subjects.

### Phase synchrony and metastability

For each of the 90 brain regions, we extracted the phases of the band-pass filtered BOLD-fMRI signals for each of the 36 recordings^64^. The phases were obtained by applying the Hilbert transform to the filtered time series, which results in the associated analytic narrowband signal, *a*(*t*). The analytic signal *a*(*t*) of a signal *x*(*t*) is defined as *a*(*t*) = *x*(*t*) + *i* ⋅ *H*[*x*(*t*)], where *i* is the imaginary unit and *H*[*x*(*t*)] denotes the Hilbert transform of *x*(*t*).

We quantify the global level of synchronisation between the nodes across time with the Kuramoto order parameter, *R*(*t*), a measure of phase locking, given by:

3 $$R(t)=|\frac{1}{n}{\sum }_{k=1}^{n}{e}^{i{\varphi }_{k}(t)}|,$$

where *n* is the total number of nodes and *φ*_*k*_(*t*) the instantaneous phase of the narrowband signal at node *k*. Thus, the Kuramoto order parameter measures the modulus of the average phase of the system at each time point and takes values from 0 to 1, where 0 represents complete phase asynchrony and 1 the completely synchronised case implying phase-locked behaviour with a phase difference of 0.

Then, to obtain measures on the group level, we calculated the temporal average and standard deviation of the Kuramoto order parameter per subject and subsequently averaged these measures within groups. Thus, we obtained for each vigilance group two measures: the group average of the mean and of the standard deviation over time of the Kuramoto order parameter, which we termed synchrony and metastability^67, 68^, respectively. The synchrony represents the global, temporally averaged level of synchronisation between all the nodes of the system, whereas the metastability gives us information about the temporal variation of the synchronisation level.

### Hopf computational whole-brain model

The computational whole-brain model is based on the 90 coupled brain areas or nodes, containing cortical and subcortical regions, derived from the AAL parcellation explained above. The local nodes are coupled through the underlying structural connectivity matrix *C*_*ij*_, obtained through DTI based tractography, which contains the fiber densities between all pairs of brain areas (Fig. 2). The structural connectivity matrix has been scaled to a maximum value of 0.2, following Deco *et al*.^26^, leading to a reduced parameter space to search for the optimal parameter combination. The resting brain dynamics emerging through the interactions of the coupled local node dynamics can be simulated with the Hopf whole-brain model. For this, the local dynamics in each brain area are simulated by the normal form of a supercritical Hopf bifurcation, which is able to describe the transition from noise-induced oscillations to full sustained oscillations^26, 69^. In fact, the Hopf model has already been applied for describing EEG dynamics^70^. The dynamics of a given node *j* are described by the following complex-valued equation:

4 $$\frac{d{z}_{j}}{dt}=z(a+i{\omega }_{j})-z{|{z}_{j}|}^{2}+\beta {\eta }_{j}(t),$$

where ${z}_{j}={\rho }_{j}{e}^{i{\theta }_{j}}={x}_{j}+i{y}_{j}$, *η*_*j*_(*t*) is additive Gaussian noise, *β* = 0.04 and *ω*_*j*_ is the intrinsic node frequency, which lies in the 0.04–0.07 Hz band. These frequencies were estimated directly from the data as the peak frequency of the filtered BOLD signals for each individual brain node averaged over participants within one group. This normal form has a supercritical Hopf bifurcation at *a = *0, meaning that for *a* < 0 there exists a stable fixed point at *z*_*j*_ = 0 which here corresponds to a low activity noisy state due to the additive Gaussian noise, and for *a* > 0 the local dynamics shows a stable limit cycle oscillation with frequency ${f}_{j}=\frac{{\omega }_{j}}{2\pi }$.

The whole-brain dynamics are then described by the following set of coupled equations:

5 $$\frac{d{x}_{j}}{dt}=\frac{d\mathrm{Re}({z}_{j})}{dt}=[a-{x}_{j}^{2}-{y}_{j}^{2}]{x}_{j}-{\omega }_{j}{y}_{j}+G{\sum }_{i}{C}_{ij}({x}_{i}-{x}_{j})+\beta {\eta }_{j}(t)$$

6 $$\frac{d{y}_{j}}{dt}=\frac{d\text{Im}({z}_{j})}{dt}=[a-{x}_{j}^{2}-{y}_{j}^{2}]{y}_{j}+{\omega }_{j}{x}_{j}+G{\sum }_{i}{C}_{ij}({y}_{i}-{y}_{j})+\beta {\eta }_{j}(t)$$

The fMRI BOLD signal is simulated by the variables *x*_*j*_ for each node *j* using the Euler algorithm with a time step of $0.1\cdot (\frac{{\rm{TR}}}{{\rm{2}}})$. The parameter *G* is introduced as a global coupling factor scaling equally all anatomical connections.

### Model fitting

We performed a parameter space exploration of the whole-brain model by varying the two free model parameters: the global coupling strength *G* was varied from 0 to 3 in steps of 0.1 and the bifurcation parameter *a* from −0.5 to 0.5 in steps of 0.02, where the bifurcation parameter *a* was changed homogeneously over nodes.The simulated time series were filtered in the range of 0.04–0.07 Hz in accordance with the empirical data and also their length coincided with the duration of the empirical data recordings. Next, we estimated the FC matrix for each parameter combination with the same procedure as explained above. Subsequently we calculated the fitting between the empirical and the simulated FC matrices for the two vigilance states for each parameter combination as the Euclidean distance between the two matrices. This resulted in one fitting value for each parameter combination per vigilance state. The whole simulation procedure was performed 50 times under exactly the same conditions, over which the results were then averaged. This was done in order to minimise the random effects caused by the Gaussian noise introduced into the model. In order to compare the two states, the minimum distance was calculated for each *G* from 0.2 onward and the according *a* was determined for each of the minima. Then, we computed the difference between the awake and the sleep state by subtracting the bifurcation parameter values (in concordance with the optimal fit for each *G*) of the sleep state minus the awake state. The resulting curve was finally compared to the curves based on the surrogate data sets. The same procedure was performed for each *a*: the minimum distance was determined for each *a* and the differences between the two states were computed.

As a next step the synchrony and metastability were computed for the whole parameter space (with *G* and *a* varying as above) based on the filtered simulated time series by applying the same procedure as on the empirical data. To compare again the two vigilance states with each other, we determined for each global coupling value *G* the according bifurcation parameter value *a*, where the simulated synchrony and metastability, respectively, was closest to the empirical one.

### Effective connectivity analysis

The effective connectivity matrix is based on the existing anatomical connections, which were obtained through the DTI fiber tractography as explained above. The general idea of the effective connections between different brain regions is here an update of the existing synaptic weights taking into account the dynamics of the whole-brain model fitted to the empirical data.

For the computation of the effective connectivity (EC) matrix we used the structural connectivity (SC) matrix as a primer. Since we took into account only the existing anatomical connections, meaning only the non-zero entries of the SC matrix, we added very small non-zero entries (Matlab machine epsilon: 2^−52^) compared to the smallest SC non-zero entry (~8.4⋅10^−5^) to the diagonal of the interhemispheric connections, since these are known to be missed by DTI based tractography^71^. Next, as a first step of the fitting procedure, the SC matrix was weighted with the fraction between the empirical and the simulated synchrony by applying the Hopf model in order to simulate the BOLD signals as done before. For the model simulations regarding the EC calculation the global coupling parameter and bifurcation parameter were fixed (*G* = 1, *a* = 0). The weighting procedure of the SC matrix was performed as long as the difference between the empirical synchrony and the simulated one was not smaller than 0.1. This weight adjustment was realised in order to bring the SC matrix closer to the final EC estimate and to avoid an exhaustive number of iteration steps in the main EC estimating procedure, which we explain in the next paragraph.

We estimated the EC for each vigilance state using a gradient descent algorithm. The previously updated SC matrix was iteratively adjusted to minimise the Euclidian distance between the empirical FC matrix (‘FCemp’) and the FC matrix predicted by the model (‘FCmodel’) through simulations of the network activity. Specifically, each iteration is given by:

7 $${{\rm{SC}}}_{ij}^{{\rm{new}}}={{\rm{SC}}}_{ij}^{{\rm{old}}}+\alpha ({{\rm{FCemp}}}_{{\rm{ij}}}-{{\rm{FCmodel}}}_{{\rm{ij}}}),$$

where *α* is the learning rate (*α* = 0.01) and (*i*, *j*) are the non-zero links of the original SC. We iterated this algorithm while the current Euclidian distance between the empirical and model values was smaller than the one obtained in the previous iteration step. We stopped the re-estimations after 200 iterations. After this procedure, the effective connectivity was given by the last updated SC matrix. Again, as in the general parameter space exploration of the model, the complete procedure for calculating the effective connectivity was performed 50 times with the exact same starting conditions. As before, the reasoning behind this is to minimise the random effects caused by the Gaussian noise present in the model.

In order to assess the differences between the EC matrices in both modalities and to investigate whether the differences are of global or rather local nature, we calculated the node strength of each node of the EC matrix^72^. Next, we calculated the mean and the standard deviation over the node strength and to test for significance we did the same for the surrogate data sets. We subtracted the mean of the sleep condition from the mean of the awake state and did again the same for the surrogate data, which gives us a global difference measure. In order to determine local differences, we computed the number of nodes displaying higher node strength during wakefulness than during sleep, and again, for significance testing, we performed the same computation on the surrogate data.

### Effective connectivity model validation

In order to validate the procedure for computing the effective connectivity between different brain areas a simple model validation simulation was performed. First, the EC matrix was calculated as described above by updating the weights of the anatomical connections by fitting the FC_model_ and the FC_emp_ in each iteration step. As a second step the same procedure was repeated, with the difference that the original empirical FC matrix was replaced by the simulated one resulting from the EC calculation in the first step. This is the optimal FC matrix resulting in the *n*th iteration step, where *n* = 1,…200, which displays the lowest distance to the empirical FC matrix. This optimal simulated FC matrix served as the new FC ground truth for the computation of the EC matrix. The anatomical connectivity matrix, on the other hand, was kept the same. The EC matrix obtained with this strategy should highly correlate with the original one if the EC computation procedure is reliable.

### Data availability

The datasets analysed during the current study are not publicly available due to constraints imposed by the ethics approval but are available from the corresponding author on reasonable request.

## Electronic supplementary material

Supplementary Information
