# Supplementary material for: Increased Stability and Breakdown of Brain Effective Connectivity During Slow-Wave Sleep: Mechanistic Insights from Whole-Brain Computational Modelling
Source: Sci Rep. 2017 Jul 5;7:4634. doi: 10.1038/s41598-017-04522-x (PMC5498661; doi:10.1038/s41598-017-04522-x)
Supplement: Supplementary file 1 — Supplementary Information [file 41598_2017_4522_MOESM1_ESM.pdf]

Supplementary Information

Increased stability and breakdown of brain effective connectivity during slow-wave sleep: mechanistic insights from whole-brain computational modelling

Beatrice M. Jobst, Rikkert Hindriks, Helmut Laufs, Enzo Tagliazucchi, Gerald Hahn, Adrián Ponce-Alvarez, Angus B.A. Stevner, Morten L. Kringelbach, Gustavo Deco

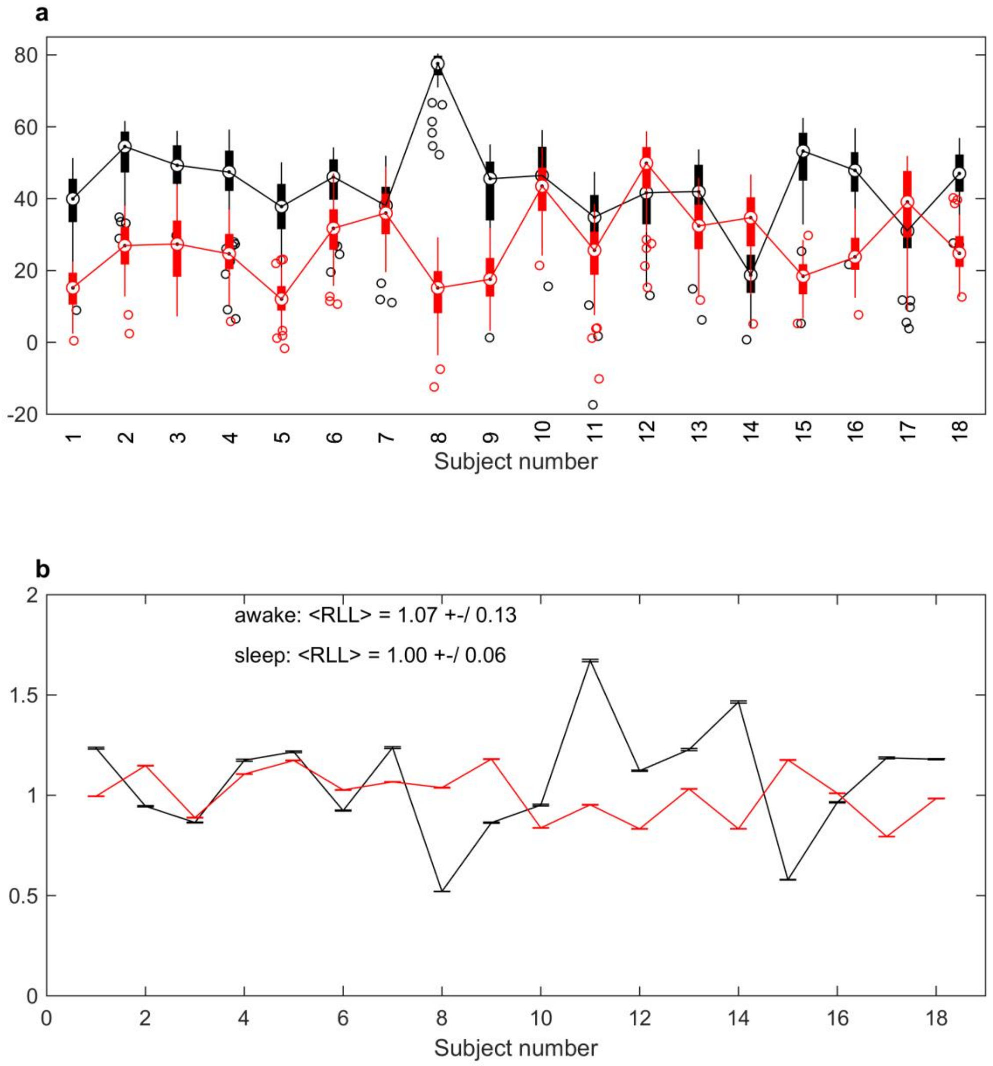

Supplementary Figure S1: Subject level data analysis. In **a** a boxplot of the node strength of individual subject-specific FC matrices is shown for awake (black) and sleep (red). It can be observed

that almost all subjects (83%) exhibited higher node strength in awake than in sleep.In **b** the average log-likelihood ratio $r_i = L(X^{(i)} \mid \Sigma_{\text{train}}) / L(X_{\text{pseudo}} \mid \Sigma_{\text{train}})$ over 5000 random samples is shown for each subject used as testset (see *Methods*), where wakefulness is represented by the black line and sleep by the red line. $\langle r_i \rangle$ is expected to be approximately 1 if the time-series of the i-th subject are indistinguishable from a random sample taken from the time-series of the remaining subjects.

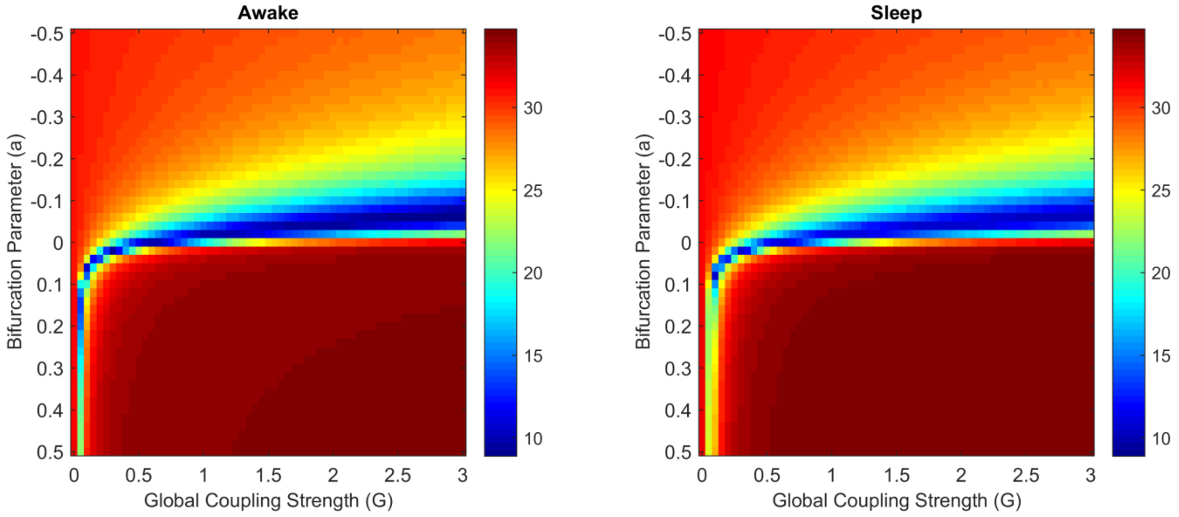

Supplementary Figure S2: Global FC fitting with same node strength. In this figure we show the Euclidean distance between FC_{model} and FC_{emp} for different values of the global coupling strength G and the bifurcation parameter *a* in awake and sleep as in Figure 3a i.+ii. with the difference that here the empirical FC matrix in sleep has been adapted such that the mean node strength was equal to the empirical FC matrix in awake. This was achieved by adding weight uniformly to all connections of the sleep FC matrix, leaving the maximum possible value at 1, until it reached the same mean node strength as the awake matrix. We can observe that there are no significant differences between the two states.

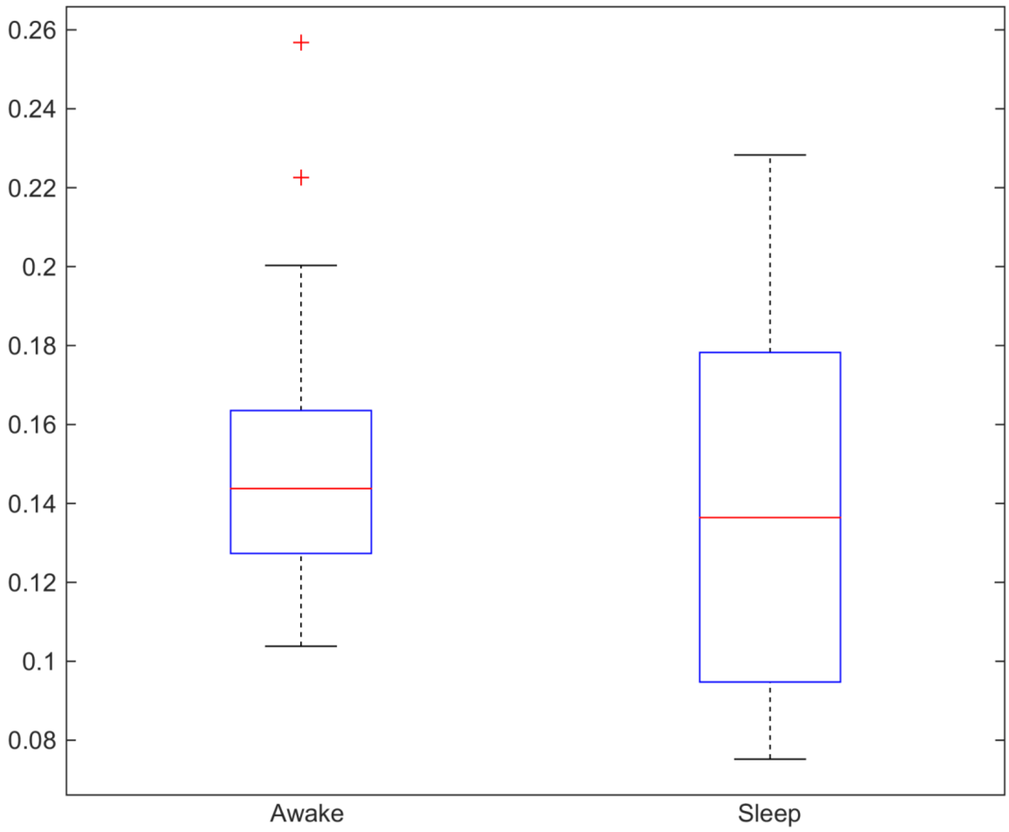

Supplementary Figure S3: Framewise displacement. Here we show a boxplot of the framewise displacement for awake and for sleep. There are no significant differences between the two brain states (see *Methods*).

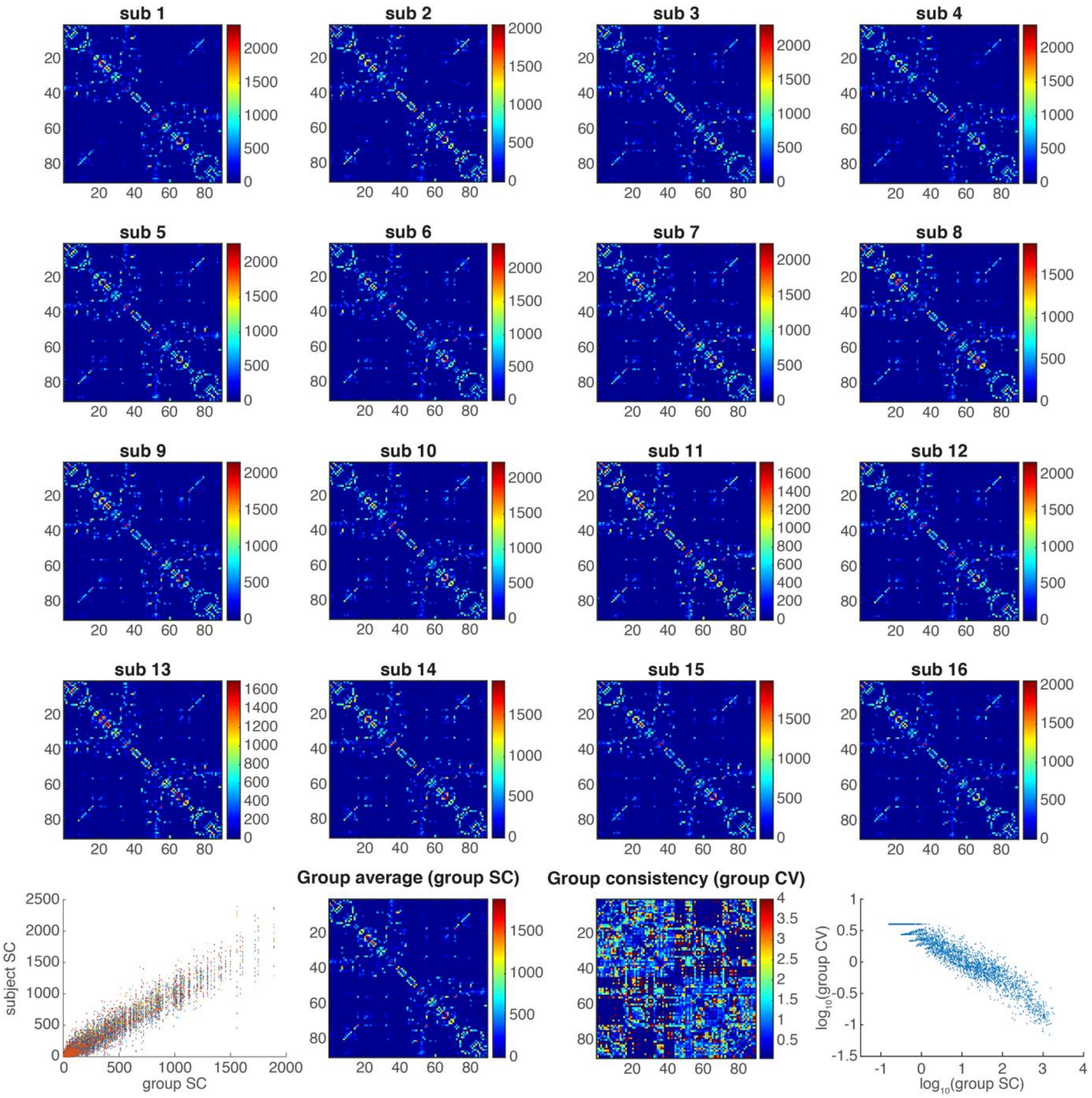

Supplementary Figure S4: Subject-specific SC analysis. In the first four rows the subject specific SC matrices are shown. In the bottom row we show (from left to right) the subject-specific SC weights as a function of the group-averaged SC matrix, which clearly shows the consistency of the group; the group average SC matrix; the group consistency matrix (std/mean; as per Roberts et al. [1]) and the group consistency as a function of group SC weights, which correspond to Fig. 1 in Roberts et al [1]. These representations demonstrate that the use of a group level SC matrix is justified.

References:

1. Roberts, J. A., Perry, A., Roberts, G., Mitchell, P. B. & Breakspear, M. Consistency-based thresholding of the human connectome. *Neuroimage* **145,** 118–129 (2017).
